# Towards environmental sustainability through the production of tailored bioplastics

**DOI:** 10.1002/2211-5463.70213

**Published:** 2026-04-01

**Authors:** C. Valeria L. Giosafatto, Raffaele Porta

**Affiliations:** ^1^ Department of Chemical Sciences University of Naples Federico II Italy

## Abstract

Plastic pollution from fossil fuel–derived materials has become a pressing environmental and public health issue, driving urgent demand for sustainable alternatives. Conventional plastics can take centuries to degrade and, instead of breaking down completely, fragment into micro‐ and nanoplastics that are now found in oceans, rivers, soil and atmosphere. These particles have been detected in drinking water, food and animal tissues, raising serious concerns about their impact on human health and ecosystems. Millions of tons of plastic enter the oceans each year, forming massive debris patches that accumulate along coastlines. Many organisms mistakenly ingest or become entangled in plastic, causing injury, starvation and often death. Birds, turtles, fish and marine mammals are among the most affected. On land, plastic waste clogs waterways, pollutes landscapes and overwhelms waste management systems, especially in regions lacking adequate recycling infrastructure. The development of bioplastics—materials derived from renewable sources and designed to biodegrade naturally—represents a promising path forward. Bioplastics aim to reduce dependence on fossil resources, minimize environmental persistence and offer tailored properties for specific applications. Transitioning to bio‐based alternatives is not only a scientific and technological challenge but also a crucial step towards safeguarding environmental health and ensuring a more sustainable future. In this context, this ‘In the Limelight’ issue of *FEBS Open Bio* presents five review articles focusing on the production, characterization and biodegradation of novel bioplastics from diverse renewable sources. Together, the results may contribute to reduced plastic pollution, offering a more sustainable and conscious approach to material design.

Bioplastics are defined as polymeric materials that may be bio‐based and/or bio‐degradable [1]. Unlike traditional plastics, which are derived from finite fossil resources and contribute significantly to pollution and greenhouse gas emissions, bioplastics are produced from renewable biological sources, such as starch, cellulose, proteins and polysaccharides. Global production capacity has grown from about 2.18 million tonnes in 2023–2.47 million tonnes in 2024, and is projected to reach over 5.7 million tonnes by 2029 [2]. This shift in raw material sourcing aligns with the principles of green chemistry and the circular economy, aiming to reduce environmental impact, promote resource efficiency and support waste valorization.

One of the key advantages of bioplastics is their potential for biodegradability and compostability. Under appropriate conditions, many bioplastics break down into non‐toxic components, reducing long‐term environmental impact and minimizing the burden on landfills and marine ecosystems. This property is particularly valuable in single‐use applications, (e.g., food packaging, agricultural films and disposable medical devices), where rapid degradation after use is preferred.

Moreover, bioplastics can be engineered to meet specific performance criteria, including mechanical strength, barrier properties, thermal resistance and optical clarity. This versatility allows them to compete with conventional plastics across a wide range of industrial sectors. Importantly, bioplastics do not inherently compromise on quality or durability. With careful formulation and processing, they can be tailored to exhibit properties comparable to or even superior to those of traditional polymers.

In fact, in their article, Repinc et al. [3] highlight the potential of bio‐based polymers in advancing sustainability across various sectors and address the existing challenges and opportunities in their development, production and application. The authors point out the importance of ensuring human health and environmental hazard assessments by incorporating principles like a ‘Safe and Sustainable by Design’ approach and assessing the ‘product's life cycle’.

In parallel, the review by Rosini et al. [4] summarizes recent advances in enzymatic degradation of oil‐derived and bio‐based polyesters. The article discusses key developments, including novel high‐throughput screenings, computational workflows for improving synthetic plastic hydrolases (such as those for polyethylene terephthalate), the *de novo* design of biocatalysts and microbial platforms for enzyme‐embedded, self‐biodegrading bioplastics.

More specifically, Pustak and Maršavelski focus on the current understanding of how polymer structure and thermal state govern enzymatic degradability, with emphasis on semicrystalline architectures and state‐dependent accessibility. Enzymatic attack preferentially begins in amorphous regions, resulting in characteristic biphasic behaviour as amorphous domains erode faster than crystalline regions. This leads to crystallinity enrichment, which subsequently slows down the degradation process [5].

However, to ensure application across different industrial sectors, it is essential to investigate the properties of bio‐based materials. As a matter of fact, recent developments in the preparation of different tailored bioplastics—highlighting the distinct properties of each type of material according to the polymers of origin—are reported by Giosafatto et al. [6]. Special attention is given to the film forming properties, barrier functionality, thermal stability and compatibility with different additives, supported by recent empirical findings. The authors focus on biopolymers extracted from renewable sources of animal and vegetal origin, such as carbohydrates and proteins found in different agricultural and industrial by‐products. Furthermore, by integrating natural extracts and industrial by‐products into biopolymer matrices, this review contributes to the advancement of sustainable materials and supports the transition towards greener packaging solutions.

Finally, Gallo et al. [7] provide a comprehensive overview of the metabolic pathways, structural properties and emerging technological innovations that are shaping the next generation of bioplastics, with a particular focus on polyhydroxyalkanoates.

In conclusion, all five reviews in this special issue collectively contribute to the advancement of sustainable materials science and provide a foundation for future industrial applications. Continued efforts should focus on scaling production and improving the technological and biological features of new ecofriendly materials. Moreover, interdisciplinary collaboration and supportive policy frameworks will be essential to facilitate the transition from laboratory innovation to commercial adoption. By bridging scientific innovation with ecological awareness, this ‘In the Limelight’ issue supports the broader goal of reducing plastic pollution and promoting a more sustainable future for novel bioplastics technologies.

## Conflict of interest

The authors declare no conflict of interest.

## Author contributions

CVLG and RP wrote the article.
